# Vitamins and *Helicobacter pylori*: An Updated Comprehensive Meta-Analysis and Systematic Review

**DOI:** 10.3389/fnut.2021.781333

**Published:** 2022-01-18

**Authors:** Xianlei Cai, Xueying Li, Yangli Jin, Miaozun Zhang, Yuan Xu, Chao Liang, Yihui Weng, Weiming Yu, Xiuyang Li

**Affiliations:** ^1^Department of Gastrointestinal Surgery, The Lihuili Affiliated Hospital, Ningbo University, Ningbo, China; ^2^Department of Gastroenterology, Ningbo First Hospital, Ningbo, China; ^3^Department of Ultrasound, Ningbo Yinzhou No.2 Hospital, Ningbo, China; ^4^Department of Epidemiology & Biostatistics, Center for Clinical Big Data and Statistics, Second Affiliated Hospital, College of Medicine, Zhejiang University, Hangzhou, China

**Keywords:** vitamins, helicobacter pylori, meta-analysis, systematic review, relationship

## Abstract

**Background:**

Over recent decades, epidemiological studies have shown relationships between vitamins and *Helicobacter pylori* (*H. pylori*) infection and eradication, but the results are controversial.

**Methods:**

A comprehensive meta-analysis and systematic review were conducted to clarify the relationships between common types of vitamins and *H. pylori*. We applied meta-regression, subgroup analysis and sensitivity analysis to obtain available evidence. Articles published from January 1991 to June 2021 in PubMed, EMBASE, and the Cochrane Library were searched.

**Results:**

In total, we identified 48 studies. The results indicate that *H. pylori* -positive patients had lower serum vitamin B_12_ [standardized mean difference (SMD) = −0.30; 95% confidence interval (CI): −0.53 – −0.08], folate (SMD = −0.69; 95% CI: −1.34 – −0.04), vitamin C (SMD = −0.37; 95%CI: −0.57 – −0.18) and vitamin D (SMD = −0.34; 95% CI: −0.49 – −0.18) levels than *H. pylori*-negative patients. Patients in which *H. pylori* had been successfully eradicated had higher serum vitamin D levels (SMD = 1.37; 95% CI: 0.37–2.38) than in patients in which eradication had been unsuccessful. The serum vitamin B_12_ levels of *H. pylori*-positive patients improved after successful *H. pylori* eradication therapy (SMD = 1.85; 95% CI: 0.81–2.90), and antioxidant vitamin supplementation to an *H. pylori* eradication regimen improved the eradication rate (risk ratio = 1.22; 95% CI: 1.02–1.44 for per-protocol analysis; risk ratio = 1.25; 95% CI: 1.06–1.47 for intention-to-treat analysis).

**Conclusions:**

*H. pylori* infections decrease the serum levels of several types of vitamins, eradication of *H. pylori* could rescue its adverse effects, and antioxidant vitamin supplementation may improve the *H. pylori* eradication rate.

**Systematic Review Registration:**

identifier: CRD42021268127.

## Introduction

*Helicobacter pylori* (*H. pylori*) is a gastric gram-negative, spiral-shaped microaerophilic pathogen (1). About half of the global population is infected with *H. pylori*, and the infection rate in developing countries is higher than in developed countries (2, 3). *H. pylori* is the main risk for chronic gastritis, gastric ulcer, gastric cancer and mucosa-associated lymphoid tissue–associated lymphoma (4–6). It can damage the gastric mucosa and affect the absorption of trace elements, especially vitamins (7). Vitamin deficiency can upset the internal balance of the human body and cause a variety of diseases outside of the digestive system (8–10).

Vitamins are members of a huge family. At present, there are dozens of known vitamins that may be divided into fat-soluble and water-soluble categories. The relationships between *H. pylori* infection and various vitamins have attracted attention worldwide. Some studies found that *H. pylori* infections reduce serum vitamin levels (11, 12); several studies have revealed that after *H. pylori* eradication, the serum vitamin levels increase (13, 14). Some randomized controlled trials (RCTs) found that vitamin supplementation combined with standard anti-*H. pylori* therapy increase the *H. pylori* eradication rate (15, 16). However, the results have been inconsistent.

Meta-analyses on the relationships between vitamins and *H. pylori* have been published (17–19). These studies involved one vitamin or a certain aspect of the relationships between vitamins and *H. pylori*, or the number of included studies was limited. Yang et al. (17) found vitamin D could improve the success rate of *H. pylori* eradication. However, they only identified three relevant studies to support this conclusion. Afsar et al. (18) reported a relationship between *H. pylori* infection and micronutrient (vitamin B_12_ and folate) levels in pregnant women. Nevertheless, the effects of *H. pylori* on the population excluding pregnant women were not evaluated. Li et al. (19) assessed the effects of antioxidant vitamins supplementation on the rate of *H. pylori* eradication. However, only three supporting studies were referenced in that work. In recent years, many excellent articles have been published. To update the results and obtain more credible conclusions, we conducted this systematic review and meta-analysis to evaluate the relationships more comprehensively, thereby providing a theoretical basis for clinical practice and public health policy-making.

## Methods

### Data Sources and Search Strategy

This meta-analysis was registered on PROSPERP (No. CRD42021268127) (20) and compliant with the main PRISMA statement (21). A comprehensive and systematic search was carried out for relevant studies describing relationships between vitamins and *H. pylori* in biologic and medical databases (Medline, Web of Science, Embase, Chinese Biomedical Database and Cambridge Scientific Abstracts databases). We developed a search strategy using following keywords: “vitamins,” “vitamin A,” “vitamin B,” “vitamin C,” “vitamin D,” “vitamin E,” “β-Carotene,” “retinol,” “cobalamin” “folate,” “folic acid,” “tocopherol,” “antioxidants,” “micronutritent,” and “*Helicobacter pylori*” (as shown in Supplementary Table 1). Duplicate works were collapsed into a single entry. Additionally, we scanned the reference lists of all the relevant published studies and reviews. The two blinded reviewers (Xianlei Cai and Xueying Li) selected the studies and specified the exclusion criteria.

### Inclusion and Exclusion Criteria

The inclusion criteria were as follows: (1) Observational or experimental research; (2) Comparisons of serum vitamin levels between *H. pylori*- positive and *H. pylori*- negative patients; (3) Comparisons of serum vitamin levels between successful and failed *H. pylori* eradication patients; (4) Comparisons of serum vitamin levels before and after successful *H. pylori* eradication therapy; (5) Comparisons of *H. pylori* eradication rate between antioxidant vitamin supplementation groups and controlled groups for *H. pylori* – positive patients; and (6) Original studies in English or Chinese indexed up to June 2021.

The exclusion criteria were as follows: (1) Original studies did not involve the relationships between vitamins and *H. pylori*; (3) Studies did not provide sufficient data for a meta-analysis; (4) reviews, comments, letters, and animal studies; and (5) low-quality studies [Newcastle-Ottawa scale and Cochrane collaboration's tool were used to assess the quality and bias risk for cross-sectional, cohort and random-controlled studies as described in a previous study (22)].

### Data Extraction

All the data were extracted by three researchers independently (Xianlei Cai, Xueying Li and Yangli Jin) using standardized form. The characteristics of the identified relevant works were records as follows: name of first author, year of publication, country, study design (cross-sectional, case series, cohort and RCTs), age, number of subjects, gender, type of vitamins (vitamins A, B, C, D and E) and presentation of effect magnitude [mean ± standard deviation (SD), mean ± standard error of mean (SEM), median (interquartile range), median (range), odds risk (OR), risk ratio (RR), or hazard ratio (HR) with 95% confidence interval (CI)].

### Statistical Analyses

Mean serum vitamin levels and the SDs were used in pooling analyses. If the original studies provided other estimates [mean ± SEM, median (interquartile range) or median (range)], we converted the data using a common method described by Hozo et al. (23). Because of the inconsistent units used in different studies, the pooled results were expressed in terms of standardized mean difference (SMD) with 95% CI. If the RCTs and cohort studies only provided 2 × 2 table data, we calculated the responding RRs. Additionally, RRs and 95% CIs were used to show the differences in the *H. pylori* eradication rates between vitamin supplementation and control groups.

A meta-regression was performed to examine the sources of heterogeneity from disparate types of vitamin supplementation (vitamin C or vitamin C plus vitamin E), and we identified influence factors having positive coefficients (*p* ≤ 0.05). Q-test and *I*^2^ were used to assess heterogeneity. If the results showed notable heterogeneity (*p* ≤ 0.05 and *I*^2^ > 50%), then pooled estimates were calculated using random-effects models (DerSimonian and Laird method) (24). Otherwise, fixed-effects models were used (Mantel-Haenszel method) (25). Subgroup analyses were performed to evaluate relationships between diverse types of vitamins and *H. pylori*. Forest and funnel plots were drawn and publication bias was tested by a weighted Egger and Begg's tests (26, 27). Sensitivity analyses were performed by omitting one estimate at a time to assess the relative influence of each work on pooled results. If the included estimates were less than four, then we did not carry out the meta-analysis and conducted systematic review instead. All the analyses were performed using STATA version 12.0 (StataCorp LP).

## Results

### Study Characteristics

**Figure 1:**
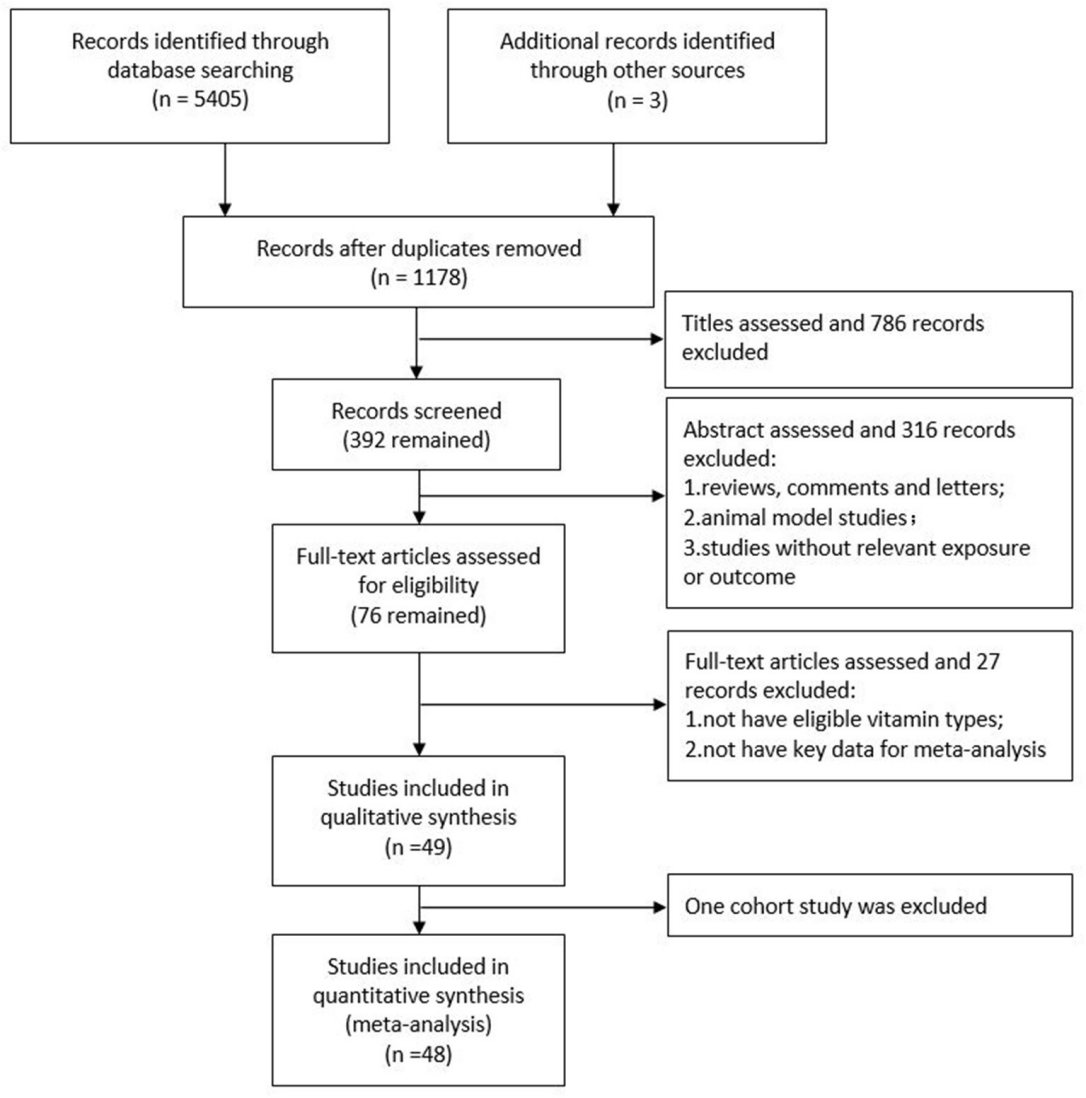
Flow diagram of the studies search process.

The flow-through of the study selection process is described using a modified PRISMA diagram (Figure 1). In total, forty-eight high quality studies (39 observational studies and 9 RCTs) with 73 independent estimates of relationships between vitamins and *H. pylori* in four fields were included in this meta-analysis and systematic review. In total, 31 studies compared serum vitamin levels between *H. pylori*- positive and - negative patients; 5 studies compared serum vitamin levels between successful and failed *H. pylori* eradication patients; 6 studies compared patient serum vitamin levels before and after successful *H. pylori* eradication therapy; and 10 studies focused on the effects of vitamin supplementation on the *H. pylori* eradication rate. Of these, three studies (28–30) considered more than one effect of *H. pylori* on vitamins. There were 173,013 participants from Turkey (12 studies), the United Kingdom (6 studies), China (4 studies), Italy (4 studies), Brazil (2 studies), Iran (2 studies), Israel (2 studies), Japan (2 studies), the USA (2 studies), and one each from Argentina, Australia, Egypt, Germany, Greece, India, Lebanon, Netherlands, Pakistan, Palestine, Poland, Saudi Arabia and Switzerland. Overall, Tables 1–**4** present the summaries of all the studies included in the meta-analysis. The original estimates reported in the articles are summarized in Supplementary Tables 2–5.

**Table 1:** Basic characteristics of studies comparing serum vitamin levels between *H*. *pylori* + groups and *H*. *pylori*- negative groups.

**Study**	**Year**	**Area**	**Design**	**Age of HP + groups**	**No. of HP + groups (male/female)**	**Age of HP - groups**	**No. of HP - groups (male/female)**	**Quality**
**Vitamin A**								
Phull et al. (31)	1998	UK	Cross-sectional	Mean 46.0 y	25 (18/7)	Mean 54 y	18 (7/11)	7^*^
Zhang et al. (32)	2000	UK	Cross-sectional	19–89 y	41 (N/A)	19-89 y	27 (N/A)	7^*^
Toyonaga et al. (33)	2000	Japan	Cross-sectional	Mean 47.0 y	37 (13/24)	Mean 44.7 y	40 (14/26)	8^*^
**Vitamin B**_**12**_								
Tamura et al. (34)	2002	Japan	Cross-sectional	Mean 64 y	57 (44/13)	Mean 63 y	36 (25/11)	8^*^
Cenerelli et al. (35)	2002	Italy	Cross-sectional	Mean 54.7 y	31 (19/12)	Mean 50.9 y	42 (23/19)	8^*^
Shuval-Sudai et al. (36)	2003	Israel	Cross-sectional	Mean 52.8 y	96 (N/A)	Mean 49.2 y	37 (N/A)	7^*^
Trimarchi et al. (37)	2004	Argentina	Cross-sectional	Mean 56.8 y	8 (3/5)	Mean 62.4 y	21 (9/12)	7^*^
Oijen et al. (38)	2004	Netherlands	Cross-sectional	N/A	29 (N/A)	N/A	60 (N/A)	7^*^
Sarari et al. (11)	2008	Palestine	Cross-sectional	Mean 43.4 y	43 (24/19)	N/A	17 (N/A)	7^*^
Stettin et al. (39)	2008	Germany	Cross-sectional	Mean 50.8 y	69 (27/42)	Mean 47.3 y	21 (8/13)	8^*^
Kakehasi et al. (40)	2009	Brazil	Cross-sectional	Mean 63.7 y	34 (0/37)	Mean 62.5 y	27 (0/27)	8^*^
Gerig et al. (41)	2013	Switzerland	Cross-sectional	Mean 42.3 y	85 (21/64)	Mean 40.9 y	319 (85/234)	8^*^
Ulasoglu et al. (42)	2019	Turkey	Cross-sectional	Mean 44.8 y	213 (N/A)	Mean 44.8 y	76 (N/A)	7^*^
Surmeli et al. (43)	2019	Turkey	Cross-sectional	Mean 74.7 y	43 (11/32)	Mean 78.2 y	211 (91/120)	9^*^
Soyocak et al. (44)	2021	Turkey	Cross-sectional	Mean 46.4 y	31 (12/19)	Mean 45.2 y	19 (8/11)	8^*^
**Folate**								
Tamura et al. (34)	2002	Japan	Cross-sectional	Mean 64 y	57 (44/13)	Mean 63 y	36 (25/11)	8^*^
Cenerelli et al. (35)	2002	Italy	Cross-sectional	Mean 54.7 y	31 (19/12)	Mean 50.9 y	42 (23/19)	8^*^
Shuval-Sudai et al. (36)	2003	Israel	Cross-sectional	Mean 52.8 y	96 (N/A)	Mean 49.2 y	37 (N/A)	7^*^
Stettin et al. (39)	2008	Germany	Cross-sectional	Mean 50.8 y	69 (27/42)	Mean 47.3 y	21 (8/13)	8^*^
Gerig et al. (41)	2013	Switzerland	Cross-sectional	Mean 42.3 y	85 (21/64)	Mean 40.9 y	319 (85/234)	8^*^
Ulasoglu et al. (42)	2019	Turkey	Cross-sectional	Mean 44.8 y	213 (N/A)	Mean 44.8 y	76 (N/A)	7^*^
Surmeli et al. (43)	2019	Turkey	Cross-sectional	Mean 74.7 y	43 (11/32)	Mean 78.2 y	211 (91/120)	9^*^
Soyocak et al. (44)	2021	Turkey	Cross-sectional	Mean 46.4 y	31 (12/19)	Mean 45.2 y	19 (8/11)	8^*^
**Vitamin C**								
Banerjee et al. (28)	1994	UK	Cross-sectional	N/A	19 (N/A)	N/A	10 (N/A)	7^*^
Rokka et al. (45)	1995	USA	Cross-sectional	Mean 43.5 y	58 (30/28)	Mean 45.5 y	30 (16/14)	8^*^
Webb et al. (46)	1997	Australia	Cross-sectional	N/A	666 (N/A)	N/A	737 (N/A)	7^*^
Phull et al. (31)	1998	UK	Cross-sectional	Mean 46 y	25 (18/7)	Mean 54 y	18 (7/11)	7^*^
Rokkas et al. (47)	1999	Greece	Cross-sectional	Mean 42.0 y	30 (17/13)	Mean 42.5 y	10 (6/4)	7^*^
Jarosz et al. (48)	2000	Poland	Cross-sectional	Mean 45.5 y/39.0 y	21 (11/10) 32 (18/14)	Mean 37.5 y/41.5 y	17 (10/7) 16 (9/7)	8^*^
Toyonaga et al. (33)	2000	Japan	Cross-sectional	Mean 47.0 y	37 (13/24)	Mean 44.7 y	40 (14/26)	8^*^
Woodward et al. (49)	2001	UK	Cross-sectional	25–74 y	765 (N/A)	25–74 y	403 (N/A)	8^*^
Everett et al. (50)	2001	UK	Cross-sectional	Mean 51 y	85 (47/38)	Mean 45 y	39 (25/14)	7^*^
Annibale et al. (29)	2003	Italy	Cross-sectional	Median 47 y	30 (6/24)	Median 37 y	13 (1/12)	7^*^
Capurso et al. (51)	2003	Italy	Cross-sectional	Median 44 y	32 (5/27)	Median 37 y	13 (1/12)	7^*^
Simon (52)	2003	USA	Cross-sectional	Mean 51 y	2189 (1072/1117)	Mean 41 y	4557 (2142/2415)	9^*^
Khanzode et al. (30)	2003	India	Cross-sectional	Mean 45.4 y	37 (15/22)	Mean 48.2 y	40 (22/18)	8^*^
**Vitamin D**								
Antico et al. (53)	2012	Italy	Cross-sectional	20–80 y	21 (N/A)	20–80 y	163 (N/A)	7^*^
Gerig et al. (41)	2013	Switzerland	Cross-sectional	Mean 42.3 y	85 (21/64)	Mean 40.9 y	319 (85/234)	8^*^
Han et al. (54)	2019	China	Cross-sectional	Mean 47.1 y	496 (236/260)	Mean 48.1 y	257 (127/300)	8^*^
Surmeli et al. (43)	2019	Turkey	Cross-sectional	Mean 74.7 y	43 (11/32)	Mean 78.2 y	211 (91/120)	9^*^
Assaad et al. (12)	2019	Lebanon	Cross-sectional	Mean 39.3 y	225 (88/137)	Mean 41.9 y	235 (88/137)	8^*^
Gao et al. (55)	2020	China	Cross-sectional	Mean 12.1 m	2113 (1202/911)	Mean 12.4 m	4783 (2865/2098)	9^*^
Shafrir et al. (56)	2021	Israel	Cross-sectional	Mean 41.0	75640 (38576/37064)	Mean 42.2	74843 (37421/37422)	9^*^
**Vitamin E**								
Phull et al. (31)	1998	UK	Cross-sectional	Mean 46 y	25 (18/7)	Mean 54 y	18 (7/11)	7^*^
Zhang et al. (32)	2000	UK	Cross-sectional	19–89 y	41 (N/A)	19–89 y	27 (N/A)	7^*^
Toyonaga et al. (33)	2000	Japan	Cross-sectional	Mean 47.0 y	37 (13/24)	Mean 44.7 y	40 (14/26)	8^*^

*UK, United Kingdom; N/A, not available; y, years; m, months*;

* *The “star system” of the Newcastle–Ottawa scale*.

### Vitamin Levels Discrepancies Between *H. pylori*- Positive and - Negative Patients

The number of studies on the relationships between *H. pylori* and vitamin B_12_, folate, vitamin C and vitamin D was sufficient for a meta-analysis (Table 1, Supplementary Table 2).

**Figure 2:**
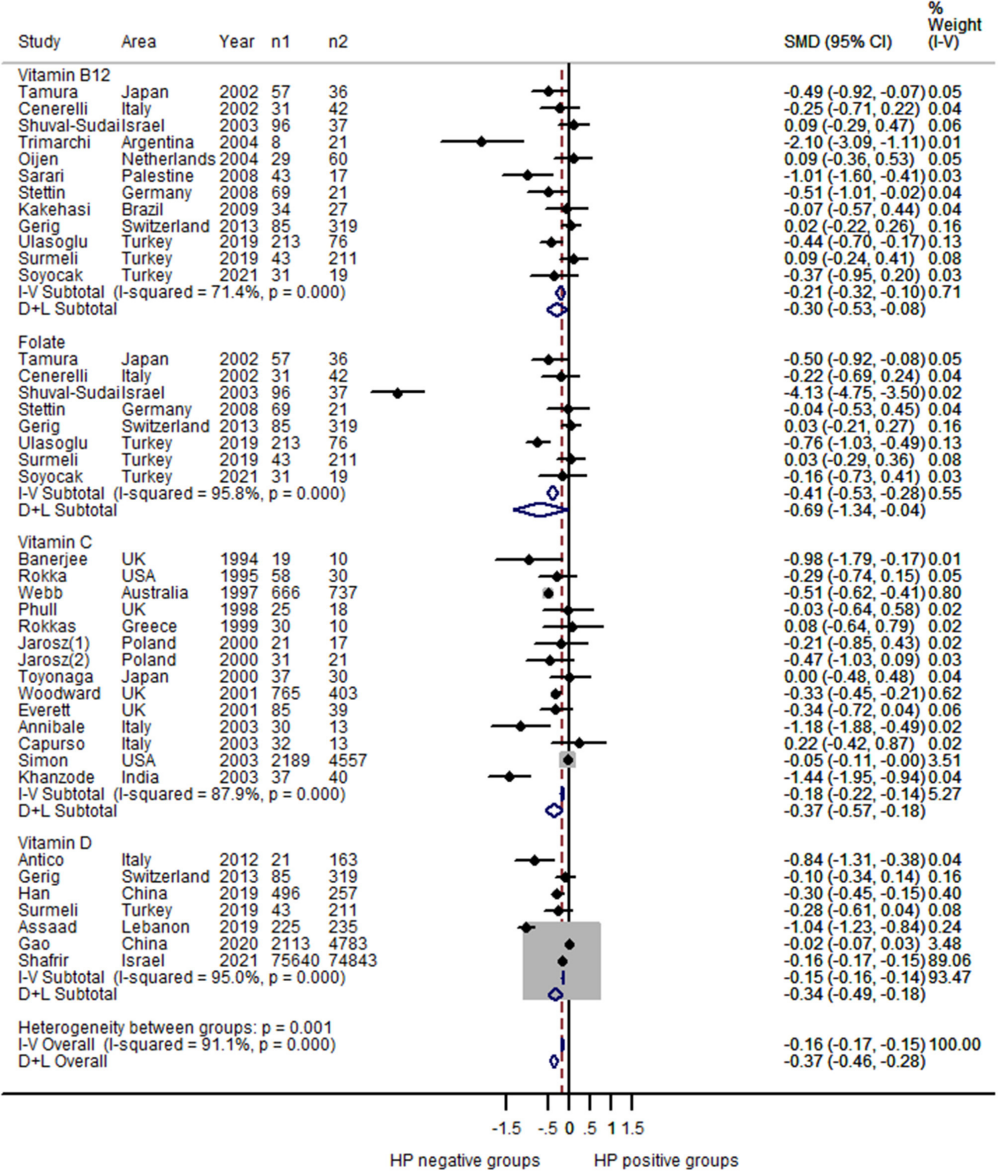
Forest plot of the meta-analysis on comparison of serum vitamin levels between *H. pylori* - positive and *H. pylori* - negative patients (n1: number of *H. pylori* – *positive* patients; n2: number of *H. pylori* – *negative* patients); *H. pylori* – *positive* patients had lower serum vitamin B12 levels than *H. pylori* – *negative* patients (SMD = –0.30; 95% CI: −0.53 – −0.08; *P* < 0.001; I^2^ = 71.4%); *H. pylori* – *positive* patients had lower serum folate levels than *H. pylori*-*negative* patients (SMD = −0.69; 95% CI: −1.34 – −0.04; *P* < 0.001; I^2^ = 95.8%); *H. pylori* – *positive* patients had lower serum vitamin C levels than *H. pylori*-*negative* patients (SMD = −0.37; 95% CI: −0.57 – −0.18; *P* < 0.001; I^2^ = 87.9%); *H. pylori* – *positive* patients had lower serum vitamin D levels than *H. pylori*-*negative* patients (SMD = −0.34; 95% CI: −0.49 – −0.18; *P* < 0.001; I^2^ = 95.0%).

**Figure 3:**
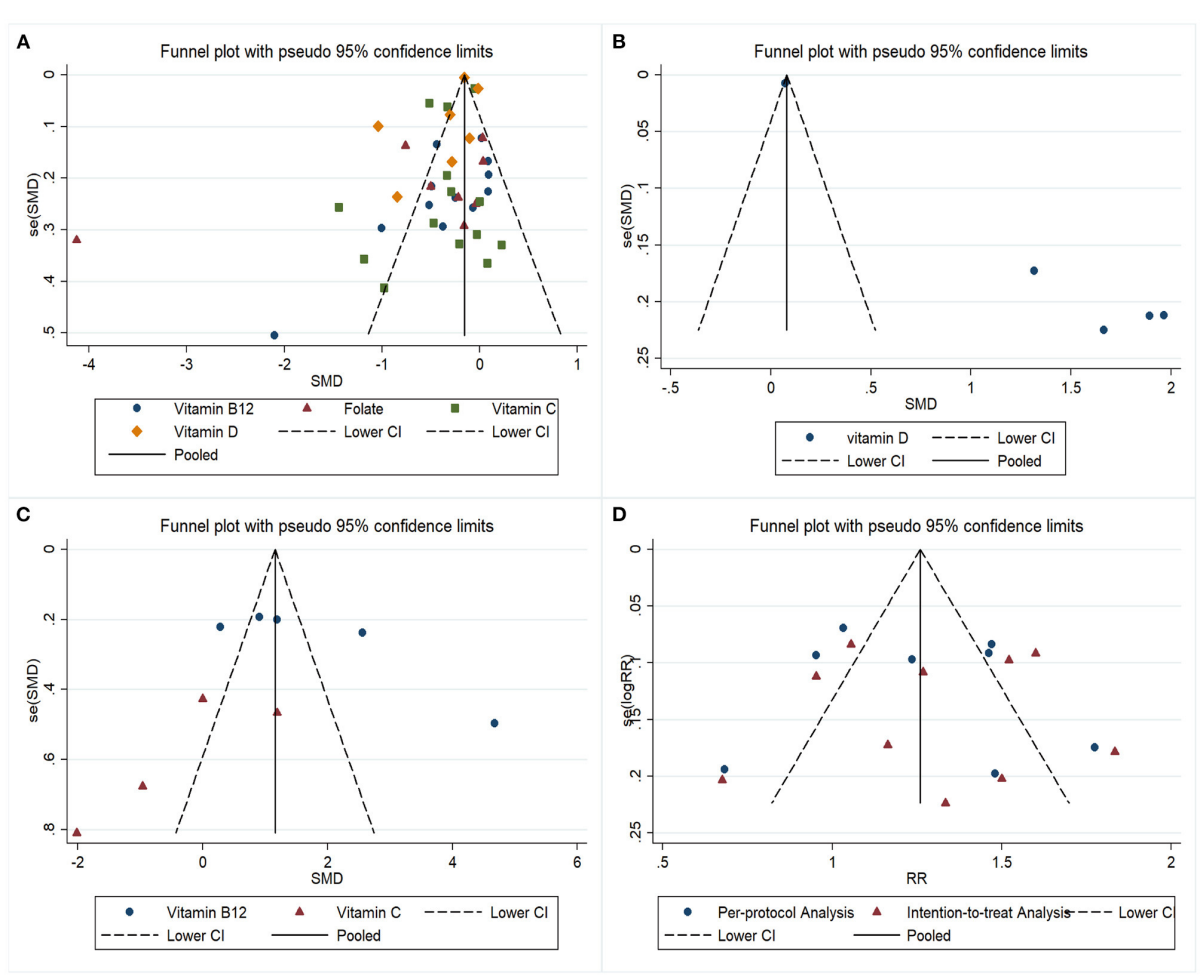
Funnel plots of the meta-analysis on relationships between *H. pylori* and vitamins: **(A)** Comparison of serum vitamin levels between *H. pylori* - *positive* and *H. pylori* - *negative* patients; **(B)** Comparison of serum vitamin levels between *H. pylori* successful and failed eradication patients; **(C)** Comparison of serum vitamin levels before and after successful *H. pylori* eradication therapy; **(D)** Effect of vitamin supplements on the *H. pylori* eradication rate.

For vitamin B_12_, 12 estimates were included in the pooled analysis. The results indicated that *H. pylori* – positive patients had lower serum vitamin B_12_ levels than *H. pylori* – negative patients (SMD = −0.30; 95% CI: −0.53 – −0.08; Figure 2), with heterogeneity (*P* < 0.001; I^2^ = 71.4%) and publication bias (Begg's test z_c_ = 2.26, *P* = 0.024; Egger's test t = 0.05; Figure 3A). The sensitivity analysis showed that the results were stable and reliable (Supplementary Figure 1A).

For folate, eight estimates were incorporated into the meta-analysis. Similarly, the results showed that *H. pylori* – positive patients had lower serum folate levels than *H. pylori*-negative patients (SMD = −0.69; 95% CI: −1.34 – −0.04; Figure 2), with obvious heterogeneity (*P* < 0.001; I^2^ = 95.8%). Publication bias was not found (Begg's test z_c_ = 0.87, *P* = 0.386; Egger's test t = 0.254; Figure 3A). The sensitivity analysis showed that the results were influenced by some positive data (Supplementary Figure 1B).

For vitamin C, 14 estimates were incorporated into the pooled analysis. The results revealed that *H. pylori* – positive patients had lower serum vitamin C levels than *H. pylori*-negative patients (SMD = −0.37; 95% CI: −0.57 – −0.18; Figure 2), with obvious heterogeneity (*P* < 0.001; I^2^ = 87.9%). There was no publication bias (Begg's test z_c_ = 0.44, *P* = 0.661; Egger's test t = 0.130; Figure 3A). The sensitivity analysis showed that the result was stable (Supplementary Figure 1C).

For vitamin D, seven estimates were included in the meta-analysis. The result also found that *H. pylori* – positive patients had lower serum vitamin D levels than *H. pylori*-negative patients (SMD = −0.34; 95% CI: −0.49 – −0.18; Figure 2), with heterogeneity (*P* < 0.001; I^2^ = 95.0%). Publication bias was not found (Begg's test z_c_ = 0.60, *P* = 0.548; Egger's test t = 0.412; Figure 3A). The sensitivity analysis revealed that the results were robust (Supplementary Figure 1D).

For vitamins A and E, only three studies were identified; therefore, we did not conduct a pooled analysis. Among these three studies, Phull et al. (31) indicated that there was no relationship between *H. pylori* infection and serum vitamin A and E levels, and their conclusion were similar to those of Zhang et al. (32) and Toyonaga et al. (33).

### Vitamin Levels Discrepancies Between Successful and Failed *H. pylori* Eradication Patients

**Figure 4:**
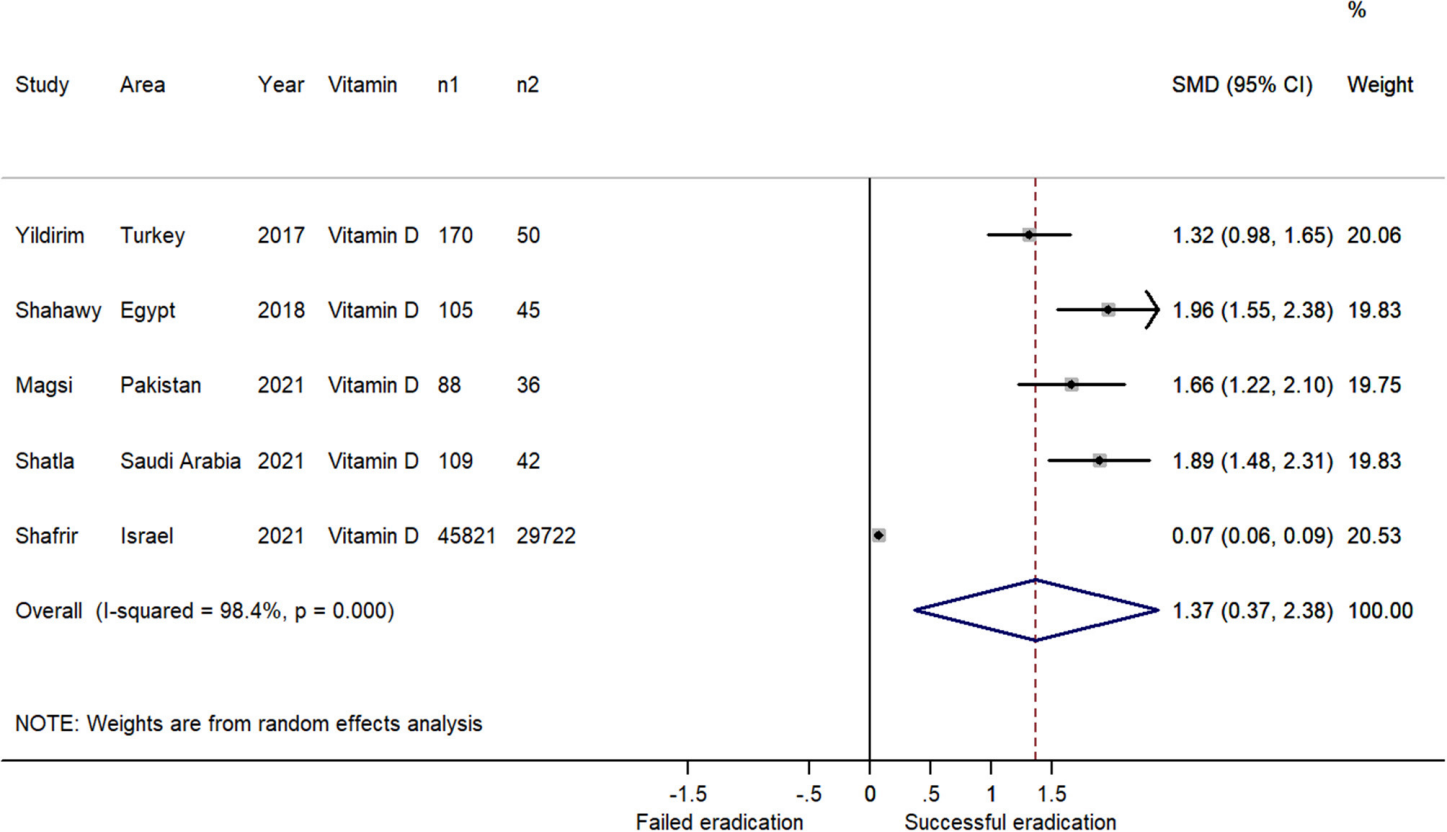
Forest plot of the meta-analysis on the comparison of serum vitamin levels between *H. pylori* successful and failed eradication patients (n1: number of successful eradication patients; n2: number of failed eradication patients); The patients with successful *H. pylori* eradication had higher serum vitamin D levels than the failed patients (SMD = 1.37; 95% CI: 0.37 – 2.38; *P* < 0.001; I^2^ = 98.4%).

Overall, five studies focusing on vitamin D level discrepancies between successful and failed *H. pylori* eradication patients were included in the meta-analysis (Table 2, Supplementary Table 3). The results indicated that the patients with successful *H. pylori* eradication had higher serum vitamin D levels than the failed patients (SMD = 1.37; 95% CI: 0.37–2.38; Figure 4), with heterogeneity (*P* < 0.001; I^2^ = 98.4%). Because all five studies reported positive estimates, the funnel plot was asymmetric (Figure 3B). We did not assess publication bias using weighted Egger test and Begg's tests owing to the insufficient numbers of estimates. The sensitivity analysis revealed that the results were robust (Supplementary Figure 2).

**Table 2:** Basic characteristics of studies comparing serum vitamin levels between the successful *H*. *pylori* eradication groups and the failed groups.

**Study**	**Year**	**Area**	**Design**	**Age of successful groups**	**No. of successful groups (male/female)**	**Age of failed groups**	**No. of failed groups (male/female)**	**Quality**
**Vitamin D**								
Yildirim et al. (57)	2017	Turkey	Cross-sectional	N/A	170 (N/A)	N/A	50 (N/A)	7^*^
Shahawy et al. (58)	2018	Egypt	Cross-sectional	18–80 y	105 (N/A)	18–80 y	45 N/A)	7^*^
Magsi et al. (2)	2021	Pakistan	Cross-sectional	18–60 y	88 (42/46)	18–60 y	36 (18/18)	8^*^
Shatla et al. (59)	2021	Saudi Arabia	Cross-sectional	N/A	109 (N/A)	N/A	42 (N/A)	7^*^
Shafrir et al. (56)	2021	Israel	Cross-sectional	N/A	45821 (N/A)	N/A	29722 (N/A)	9^*^

*N/A, not available; y, years*;

* *The “star system” of the Newcastle–Ottawa scale*.

There were no studies exploring other vitamin levels (vitamin A, B, C and E) discrepancies between successful and failed *H. pylori* eradication patients. More relevant research is recommended to clarify the relationships.

### Vitamin Level Discrepancies Before and After Successful *H. pylori* Eradication Therapy

**Figure 5:**
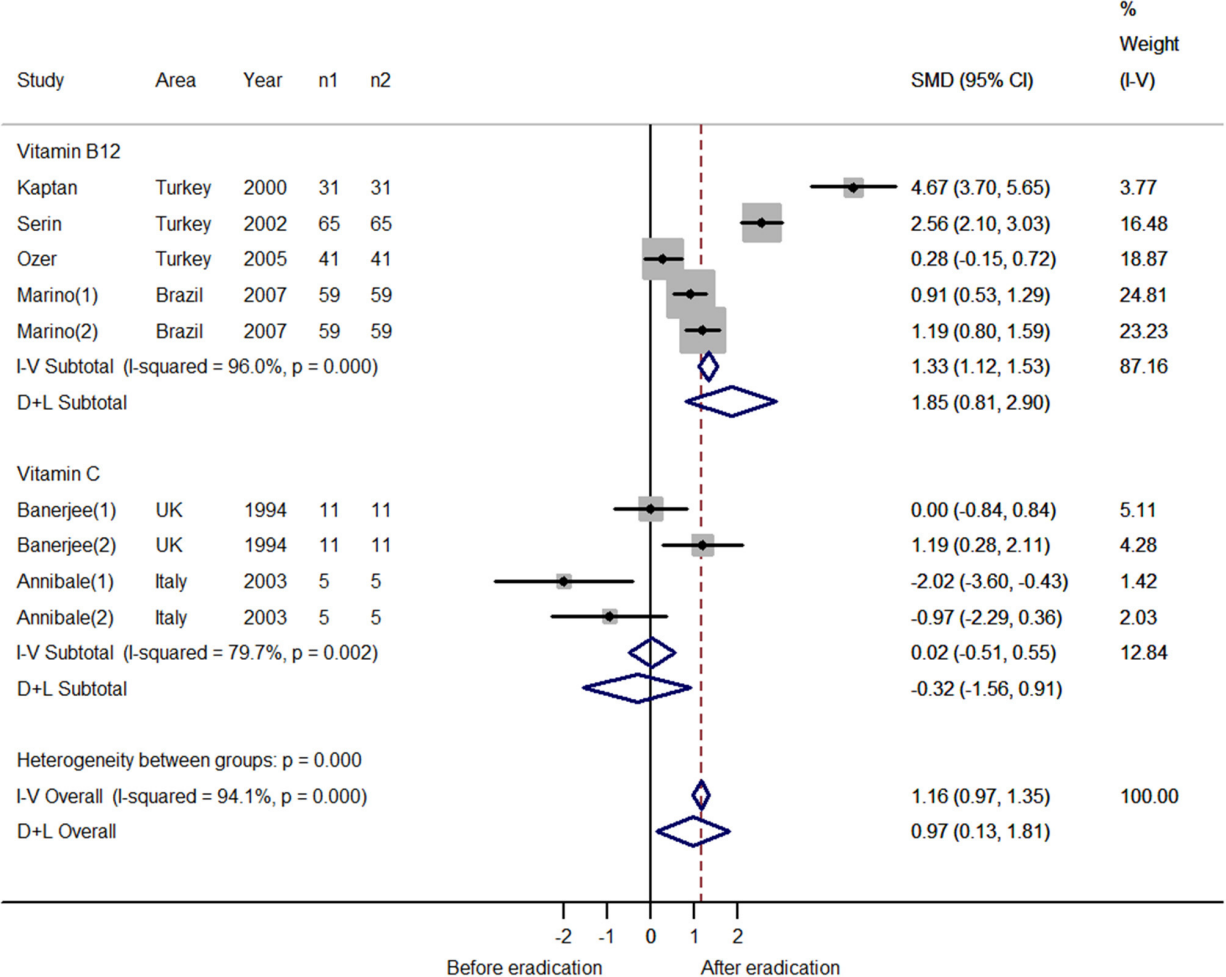
Forest plot of the meta-analysis on the comparison of serum vitamin levels before and after successful *H. pylori* eradication therapy (n1: number of patients after eradication therapy; n2: number of patients before eradication therapy); After successful *H. pylori* eradication, serum vitamin B12 increased (SMD = 1.85; 95% CI: 0.81–2.90; *P* < 0.001; I^2^ = 96.0%); *H. pylori* eradication did not increase the serum vitamin C level (SMD = −0.32; 95% CI: −1.56–0.91; *P* = 0.002; I^2^ = 79.7%).

The numbers of studies for vitamins B_12_ and C were sufficient for a meta-analysis (Table 3, Supplementary Table 4). For vitamin B_12_, five estimates were incorporated into the pooled analysis. The result indicated that after successful *H. pylori* eradication, serum vitamin B_12_ increased (SMD = 1.85; 95% CI: 0.81–2.90; Figure 5), with heterogeneity (*P* < 0.001; I^2^ = 96.0%). The funnel plot was symmetric (Figure 3C). The sensitivity analysis showed that the results were stable and reliable (Supplementary Figure 3). For vitamin C, four estimates were included in the meta-analysis. The results showed that *H. pylori* eradication did not increase the serum vitamin C level (SMD = −0.32; 95% CI: −1.56–0.91; Figure 5), with heterogeneity (*P* = 0.002; I^2^ = 79.7%). The funnel plot was symmetric (Figure 3C). Because the four estimates were extracted from two studies, we did not conduct a sensitivity analysis and the results should be interpreted cautiously.

**Table 3:** Basic characteristics of studies comparing serum vitamin levels before and after *H*. *pylori* eradication therapy.

**Study**	**Year**	**Area**	**Design**	**Age**	**No. before eradication (male/female)**	**No. after eradication (male/female)**	**Test time after eradication**	**Quality**
**Vitamin B**_**12**_								
Kaptan et al. (13)	2000	Turkey	Case series	Mean 59.5 y	31 (19/12)	31 (19/12)	3 or 6 m	8^*^
Serin et al. (60)	2002	Turkey	Case series	Mean 43 y	65 (N/A)	65 (N/A)	2–3 m	7^*^
Ozer et al. (61)	2005	Turkey	Case series	Mean 41 y	41 (N/A)	41 (N/A)	1 m	7^*^
Marino et al. (14)	2007	Brazil	Case series	Mean 72.8 y	59 (N/A)	59 (N/A)	6 m / 12 m	7^*^
**Folate**								
Kaptan et al. (13)	2000	Turkey	Case series	Mean 59.5 y	31 (19/12)	31 (19/12)	3 or 6 m	8^*^
Ozer et al. (61)	2005	Turkey	Case series	Mean 41 y	41 (N/A)	41 (N/A)	1 m	7^*^
**Vitamin C**								
Banerjee et al. (28)	1994	UK	Case series	N/A	11 (N/A)	11 (N/A)	1 m / 6 m	7^*^
Annibale et al. (29)	2003	Italy	Case series	Median 47 y	5 (N/A)	5 (N/A)	6 m	7^*^

*N/A, not available; y, years; m, months*;

* *The “star system” of the Newcastle–Ottawa scale*.

For folate, we only identified two relevant studies; therefore, a pooled analysis was not performed. Both Kaptan et al. (13) et al. and Ozer et al. (61) concluded that successful *H. pylori* eradication was not associated with an increased serum folate level.

### The Effects of Antioxidant Vitamin Supplementation on *H. pylori* Eradication

A total of 10 trials on the effects of antioxidant vitamin supplementation on *H. pylori* eradication were identified (Table 4 and Supplementary Table 4). Among them, nine studies were RCTs, and one study was cohort research. The risks of bias were summarized in Supplementary Table 6. To avoid heterogeneity derived from different study designs, we did not include the cohort research (69) in the meta-analysis. We calculated the pooled results of the per-protocol analysis and intention-to-treat analysis from RCTs.

**Table 4:** Basic characteristics of studies focusing on the effect of vitamin supplementation on *H*. *pylori* eradication rate.

**Study**	**Year**	**Area**	**Design**	**Age of group 1**	**No. of group 1 (male/female)**	**Age of group 2**	**No. of group 2 (male/female)**	**Type of vitamin supplementation**	**Method of HP eradication**
Chuang et al. (62)	2002	China	RCT	Mean 37.9 y	55 (21/34)	Mean 35.6 y	49 (19/30)	Vitamin C plus Vitamin E	Triple therapy
Everett et al. (63)	2002	UK	RCT	Mean 52 y	29 (17/12)	Mean 49 y	30 (16/14)	Vitamin C plus Vitamin E	Triple therapy
Sezikli et al. (15)	2009	Turkey	RCT	Mean 43 y	80 (24/56)	Mean 44 y	80 (28/52)	Vitamin C plus Vitamin E	Quadruple therapy
Sezikli et al. (64)	2011	Turkey	RCT	Mean 42 y	80 (25/55)	Mean 43 y	40 (15/25)	Vitamin C plus Vitamin E	Triple therapy
Sezikli et al. (65)	2012	Turkey	RCT	Mean 39.7	160 (53/107)	Mean 42.7	40 (13/27)	Vitamin C plus Vitamin E	Triple therapy
Demirci et al. (66)	2015	Turkey	RCT	Mean 40 y	100 (59/41)	Mean 41 y	100 (53/47)	Vitamin C plus Vitamin E	Triple therapy / Quadruple therapy
Kockar et al. (67)	2001	Turkey	RCT	Mean 40.0 y	30 (N/A)	Mean 38.9 y	30 (N/A)	Vitamin C / Vitamin A	Triple therapy
Chuang et al. (68)	2007	China	RCT	Mean 53.2 y	61 (N/A)	Mean 49.9 y	55 (N/A)	Vitamin C	Triple therapy
Zojaji et al. (16)	2009	Iran	RCT	Mean 43 y	150 (N/A)	Mean 45 y	162 (N/A)	Vitamin C	Triple therapy
Kaboli et al. (69)	2009	Iran	Cohort	N/A	114 (N/A)	N/A	100 (N/A)	Vitamin C	Triple therapy

*Group 1: antibiotic + vitamin groups*.

*Group 2: antibiotic groups*.

*RCT, random controlled trial; N/A, not available; y, years; UK, United Kingdom*.

**Figure 6:**
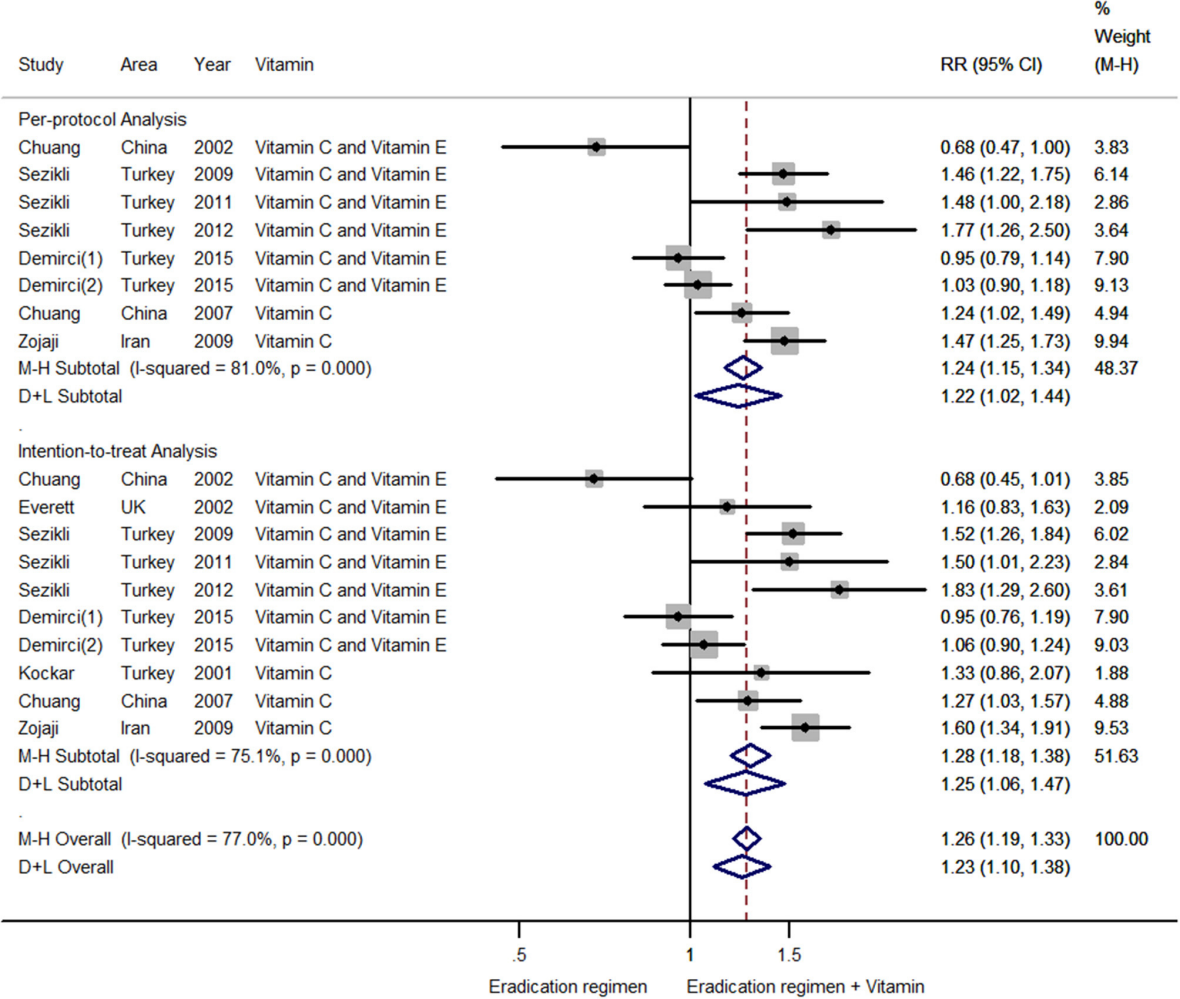
Forest plot of the meta-analysis on the effects of vitamin supplements on *H. pylori* eradication rate (n1: number of eradication regimen plus vitamin supplement group; n2: number of eradication regimen group); For the per-protocol analysis, combining antioxidant vitamin supplementation with the standard therapy increased the *H. pylori* eradication rate (RR = 1.22; 95% CI: 1.02–1.44; *P* < 0.001; I^2^ = 81.0%); For the intention-to-treat analysis, antioxidant vitamins supplementation increased the *H. pylori* eradication rate (RR = 1.25; 95%CI: 1.06–1.47; *P* < 0.001; I^2^ = 75.1%).

For the per-protocol analysis, eight estimates were included. A meta-regression was performed to assess potential sources of heterogeneity from eradication therapy (triple or quadruple therapy) and types of vitamin supplementation (vitamin C or vitamin C plus vitamin E). We found that eradication therapy and types of vitamin supplementation were not influencing factors (*P* = 0.441 for eradication therapy; *P* = 0.707 for types of vitamin supplementation). Therefore, all eight estimates were incorporated into the meta-analysis. The results indicated that combining antioxidant vitamin supplementation with standard therapy could increase the *H. pylori* eradication rate (RR = 1.22; 95% CI: 1.02–1.44; Figure 6), with heterogeneity (*P* < 0.001; I^2^ = 81.0%). There was no publication bias (Begg's test z_c_ = 0.12, *P* = 0.902; Egger's test t = 0.662), and the funnel plot was symmetric (Figure 3D). The sensitivity analysis revealed that the results were robust (Supplementary Figure 4).

For the intention-to-treat analysis, 10 estimates were included. A meta-regression was conducted, and the result revealed that eradication therapy and types of vitamin supplementation were not influencing factors (*P* = 0.832 for eradication therapy; *P* = 0.510 for types of vitamin supplementation). The results of the pooled analysis showed that antioxidant vitamins supplementation increased the *H. pylori* eradication rate (RR = 1.25; 95%CI: 1.06–1.47; Figure 6), with heterogeneity (*P* < 0.001; I^2^ = 75.1%). Publication bias was not found (Begg's test z_c_ = 0.18, *P* = 0.858; Egger's test t = 0.973; Figure 3D). The sensitivity analysis showed that the results were stable.

Only the Kockar et al. study (67) explored the effects of vitamin A supplementation on *H. pylori* eradication. They determined that vitamin A was ineffective in *H. pylori* eradication. More relevant RCTs are recommended to clear the relationship.

## Discussion

The debate over the *H. pylori*-vitamin association has been persistent. The controversy mainly focuses on the following four aspects: (1) vitamin level discrepancies between *H. pylori*-positive and -negative patients; (2) vitamin level discrepancies between successful and failed *H. pylori* eradication patients; (3) vitamin level discrepancies before and after successful *H. pylori* eradication therapy; and (4) the effects of vitamin supplementation on *H. pylori* eradication. Phull et al. (31), Zhang et al. (32) and Toyonaga et al. (33) found that *H. pylori* infection was not associated with serum vitamin A and E levels. The vitamin B_12_ results from Trimarchi et al. (37), Sarari et al. (11) and Ulasoglu et al. (42) indicated that *H. pylori* had an adverse effect on serum vitamin B_12_ levels, whereas other observational studies (35, 36, 40, 44) presented a null association between *H. pylori* and vitamin B_12_. The findings of Tamura et al. (34), Shuval-Sudai et al. (36) and Ulasoglu et al. (42) showed that *H. pylori*-negative patients had higher serum folate levels than *H. pylori*-positive patients; however, some studies (35, 39, 41, 43, 44) did not present similar results. A similar controversy exists regarding the associations between serum vitamin C or D levels and *H. pylori*. The effects of *H. pylori* eradication on serum vitamin levels have aroused extensive interest. Several studies (2, 57–59) found that patients that underwent successful *H. pylori* eradication had higher serum vitamin D levels than patients with in which eradication failed. Several studies (13, 14, 60, 61) described vitamin B_12_ level discrepancies before and after successful *H. pylori* eradication therapy; nevertheless, there were also inconsistent results. Vitamin supplementation was hypothesized to aid in *H*. *pylori* eradication. Some high-quality RCTs were performed to assess the assisting role of antioxidant vitamin supplementation on *H*. *pylori* eradication. Sezikli et al. (65) found that supplementation with vitamin C plus vitamin E increases the *H. pylori* eradication rate; whereas Chuang et al. (68) and Zojaji et al. (16) indicated that simple vitamin C supplementation increases the *H*. *pylori* eradication rate. In contrast, studies from other researchers (66, 67) showed that vitamin supplementation has no effects on the *H*. *pylori* eradication rate. Several meta-analyses had also been published. Yang et al. (17) found that 25-hydroxyvitamin D levels in *H. pylori -* negative patients were higher than in *H. pylori –* positive patients, and patients with vitamin D deficiency had lower eradication rates of *H. pylori*. Lahner et al. (70) reported a comprehensive meta-analysis in 2012 focused on the relationships between micronutrients and *H. pylori*. This study found that *H. pylori* was associated with ascorbic acid levels in gastric juice, which were increased by the eradication treatment. At present, many relevant articles have been published, and we have the opportunity to update the results and obtain reliable conclusions.

The results of the meta-analysis suggested negative effects of *H. pylori* on serum vitamin B_12_, folate, vitamin C and vitamin D levels. *Helicobacter pylori*-induced atrophic gastritis impairs stomach acidification and secretion functions and causes the malabsorption of nutrients. Vitamin B_12_ plays indispensable roles in promoting the development and maturation of red blood cells and in maintaining normal hematopoietic functions (71). A deficiency in an intrinsic factor caused by *H*. *pylori* infection would aggravate vitamin B_12_ absorption-related disorders_._ Folate participates in the metabolism of genetic materials and proteins, and it affects mammal reproduction (72). A folate deficiency may cause neural tube malformation, megaloblastic anemia, depression and malignant tumor formation (71, 73). A vitamin B_12_ deficiency may cause a folate metabolic disorder and aggravate the folate deficiency. Vitamin C and E, as vitamins with antioxidant effects, play roles in eradicating oxygen radicals and in maintaining the body's steady state (74, 75). Vitamin C may also promote the formation of tetrahydrofolic acid (75, 76). A shortage of vitamin C aggravates a folate deficiency. Therefore, the effects of *H*. *pylori* infections on human vitamin levels are not independent, and there are interactions among the various vitamins.

Our study found that patients that had undergone successful *H. pylori* eradication had higher serum vitamin D levels than patients in which *H. pylori* eradication failed. We speculated that vitamin D is a protective factor for *H*. pylori. In terms of mechanism, a combination of vitamin D and the vitamin D receptor may activate immune responses and participate in the anti-*H*. *pylori* process (77, 78). Yang et al. (17) also performed a related meta-analysis, but they only included three papers; consequently, the limited number of studies was not suitable for a pooled analysis. Our work included five relevant studies, including the study of Shafrir et al. (56) that covered data from more than 70,000 patients, which improved the reliability of the results. Apart from those on vitamin D, we did not find any studies that explored the relationships between other vitamins and the success of *H*. *pylori* eradication. Further research is needed to investigate the differences in other vitamin levels between patients with successful *H*. *pylori* eradication compared with those that failed.

This work revealed that serum vitamin B_12_ levels in patients after *H*. *pylori* eradication were significantly higher than those before *H*. *pylori* eradication. We reaffirmed the adverse effects of *H*. *pylori* on vitamin B_12_. For patients with a vitamin B_12_ deficiency, aggressive *H*. *pylori* eradication therapy and additional vitamin B_12_ supplementation are necessary. For vitamin C, this meta-analysis did not produce a statistically significant result. However, the four estimates included in this study were from only two reports. Thus, there were too few relevant studies included, and the results should be interpreted cautiously.

The pooled results of the RCTs showed that both the per-protocol analysis and intention-to-treat analysis suggested that antioxidant vitamin supplementation could improve the eradication rates of *H*. *pylori* of standard regimens. In the selection of standard eradication regimens, Kockar et al. (67), Chuang et al. (68) and Zojaji et al. (16) chose vitamin C for additional supplementation, whereas other studies used vitamin C combined with vitamin E for supplementation. Although both vitamin C and vitamin E have antioxidant effects, we were still concerned that inconsistent supplementation selection would introduce bias. Moreover, except the studies of Sezikli et al. (15) and Demirci et al. (66), which adopted the quadruple regimen, other studies adopted the triple regimen. The choices of eradication therapy (triple or quadruple therapy) also could introduce bias. We used a meta-regression to eliminate the possible bias caused by different kinds of vitamin supplementation and different eradication schemes from the statistical field, and then, we conducted pooled analyses to improve the reliability of the results. The meta-analysis of Li et al. (19) found that supplementation with antioxidant vitamins did not benefit the eradication rate. However, this analysis included only three studies. Although Ochoa et al. (79) also conducted a meta-analysis on a similar topic in 2018, there were errors and shortcomings in the data extraction and chart construction. Moreover, the above two articles only performed intention-to-treat analyses of the data. We believed that the per-protocol analysis should not be ignored in the RCTs. The meta-analysis of Yang-Ou et al. (80) focused on the effects of antioxidants on *H*. *pylori* eradication. However, this study combined antioxidant vitamins with other antioxidant (curcumin and cranberry). Our study incorporated intention-to-treat and per-protocol analyses and updated the results. Interestingly, the cohort study by Kaboli et al. (69) found that vitamin C supplementation reduces the dosage of clarithromycin. Li et al. (81) revealed that the *H*. *pylori* treatment and vitamin supplementation reduced the incidence of gastric cancer.

A complete and comprehensive search is necessary to find all the published data relevant to the meta-analysis. In addition to the usual English databases (Medline, Web of Science and Embase), we also searched for Chinese studies using the Chinese Biomedical Database and for conference papers using the Cambridge Scientific Abstracts databases to identify suitable studies. Although the search results did not ultimately expand the number of relevant articles, the processes remained essential. Unfortunately, it was hard for us to search manuscript with other languages, which might have resulted in the exclusion of some suitable articles. At last, we identified 48 relevant articles in this meta-analysis. We ran a better selection of studies and the number of included articles was larger than previous meta-analysis.

The heterogeneity of the selected studies cannot be ignored. The different methods of vitamin detection may result in variations in the results. The different areas, different numbers of patients enrolled in each study, some positive data and large amounts of included estimates may have increased the heterogeneity. Because the number of included studies was limited, it was hard for us to conduct subgroup analyses to reduce the influence of heterogeneity. Nevertheless, we performed meta-regression and sensitivity analyses to reduce the effects of heterogeneity on the credibility of the conclusions. The results of the meta-regression helped us eliminate the possibility that heterogeneity was a result of different eradication methods or vitamin supplementations. The sensitivity analyses helped us eliminate the influence of some positive data on the conclusions. In addition to the meta-regression and sensitivity analyses, we used random-effects models to establish relationships among the variables with high heterogeneity. Therefore, this work is appropriate to provide evidence, but the conclusions should be interpreted cautiously.

The study provides a comprehensive analysis of the interrelations between *H*. *pylori* and the most common vitamins from four aspects. Nevertheless, this work has some shortcomings. First, Turkish scholars have done a great deal of work on the effects of vitamin supplementation on *H*. *pylori* eradication. However, the contributions of researchers from other countries are limited. Different races and dietary habits may influence the results. It is difficult to conduct subgroup analyses to address this issue. Second, quadruple anti-*H*. *pylori* therapy is recommended currently. Some studies included in the work still used the triple approach. Moreover, the dose of vitamin supplementation is also controversial. Thus, the results should be interpreted cautiously. More RCTs with large samples focusing on the effects of vitamin supplementation on *H*. *pylori* eradication are needed to confirm our results and explore the appropriate dose.

## Conclusions

In summary, this meta-analysis demonstrates that *H*. *pylori* infections can reduce the serum levels of several vitamins. The eradication of *H*. *pylori* rescues its adverse effects. Antioxidant vitamin supplementation may increase the rate of *H*. *pylori* eradication. Aggressive *H*. *pylori* eradication therapy is necessary, and the advantages of multivitamin supplementation for *H*. *pylori*-positive patients outweigh the disadvantages.

## Data Availability Statement

The original contributions presented in the study are included in the article/Supplementary Material, further inquiries can be directed to the corresponding authors.

## Author Contributions

XC, WY, and XiL conceived and designed the study. XC and XuL acquired and analyzed the data. XC, YJ, MZ, YX, YW, and CL interpreted the data and drafted the manuscript. XiL and WY reviewed and corrected the manuscript. All authors approved the final version to be published.

## Funding

This work was supported by the Soft Science Key Project of the Science and Technology Department of Zhejiang Province (2019C25009 and 2022C25040), the Key Project of Social Science Planning in Hangzhou City (hzjz20180110), the Soft Science Key Project of Hangzhou Municipal Science Committee (20160834M03), the Ningbo Clinical Medicine Research Center Project (2019A21003), Zhejiang Provincial Natural Science Foundation (LQ21H160013), and Zhejiang Provincial Medical and Health Science and Technology Project (2022493920). This study also received funding from Chiatai Qingchunbao Pharmaceutical Co. Ltd. The funder was not involved in the study design, collection, analysis, interpretation of data, the writing of this article or the decision to submit it for publication.

## Conflict of Interest

The authors declare that the research was conducted in the absence of any commercial or financial relationships that could be construed as a potential conflict of interest.

## Publisher's Note

All claims expressed in this article are solely those of the authors and do not necessarily represent those of their affiliated organizations, or those of the publisher, the editors and the reviewers. Any product that may be evaluated in this article, or claim that may be made by its manufacturer, is not guaranteed or endorsed by the publisher.
