# Supplementary material for: Vitamins and Helicobacter pylori: An Updated Comprehensive Meta-Analysis and Systematic Review
Source: Front Nutr. 2022 Jan 18;8:781333. doi: 10.3389/fnut.2021.781333 (PMC8805086; doi:10.3389/fnut.2021.781333)
Supplement: Supplementary file 5 [file Table_1.DOCX]

Supplementary Table 1 Search strategy of Medline

| # | Searches |
| --- | --- |
| 1 | vitamins |
| 2 | vitamin A |
| 3 | vitamin B |
| 4 | vitamin C |
| 5. | vitamin D |
| 6. | vitamin E |
| 7. | β-Carotene |
| 8. | retinol |
| 9. | cobalamin |
| 10. | folate |
| 11. | folic acid |
| 12. | tocopherol |
| 13. | antioxidants |
| 14. | micronutritent |
| 15. | Helicobacter pylori |
| 16. | Helicobacter pylori infection |
| 17. | Helicobacter pylori eradication |
| 18. | 1 and 15 |
| 19 | 1 and 16 |
| 20 | 1 and 17 |
| 21 | 2 and 15 |
| 22 | 2 and 16 |
| 23 | 2 and 17 |
| 24 | 3 and 15 |
| 25 | 3 and 16 |
| 26 | 3 and 17 |
| 27 | 4 and 15 |
| 28 | 4 and 16 |
| 29 | 4 and 17 |
| 30 | 5 and 15 |
| 31 | 5 and 16 |
| 32 | 5 and 17 |
| 33 | 6 and 15 |
| 34 | 6 and 16 |
| 35 | 6 and 17 |
| 36 | 7 and 15 |
| 37 | 7 and 16 |
| 38 | 7 and 17 |
| 39 | 8 and 15 |
| 40 | 8 and 16 |
| 41 | 8 and 17 |
| 42 | 9 and 15 |
| 43 | 9 and 16 |
| 44 | 9 and 17 |
| 45 | 10 and 15 |
| 46 | 10 and 16 |
| 47 | 10 and 17 |
| 48 | 11 and 15 |
| 49 | 11 and 16 |
| 50 | 11 and 17 |
| 51 | 12 and 15 |
| 52 | 12 and 16 |
| 53 | 12 and 17 |
| 54 | 13 and 15 |
| 55 | 13 and 16 |
| 56 | 13 and 17 |
| 57 | 14 and 15 |
| 58 | 14 and 16 |
| 59 | 14 and 17 |

The same strategy for other databases
