# Supplementary material for: Vitamins and Helicobacter pylori: An Updated Comprehensive Meta-Analysis and Systematic Review
Source: Front Nutr. 2022 Jan 18;8:781333. doi: 10.3389/fnut.2021.781333 (PMC8805086; doi:10.3389/fnut.2021.781333)
Supplement: Supplementary file 6 [file Table_2.DOCX]

Supplementary Table 2 Original data of the studies comparing serum vitamin level between HP+ groups and HP- negative group

| Study | Year | Area | Estimates presentation | No. of HP + groups | Original data of HP + groups | No. of HP - groups | Original data of HP - groups |
| --- | --- | --- | --- | --- | --- | --- | --- |
| **Vitamin A** |  |  |  |  |  |  |  |
| Phull | 1998 | UK | Median (IQR) | 25 | 74.1 (68.4-105.3) μg/dl | 18 | 81.3 (62.3-106.3) μg/dl |
| Zhang | 2000 | UK | Median (IQR) | 41 | 0.3 (0.2-0.4) μmol/l | 27 | 0.2 (0.2-0.3) μmol/l |
| Toyonaga | 2000 | Japan | Mean ± SD | 37 | 28 ± 16.7μg/dl | 30 | 27.8 ± 23.9 μg/dl |
| **Vitamin B_12_** |  |  |  |  |  |  |  |
| Tamura | 2002 | Japan | Mean ± SD | 57 | 630 ± 222 pg/ml | 36 | 747 ± 259 pg/ml |
| Cenerelli | 2002 | Italy | Mean ± SD | 31 | 451.5 ± 188.4 pg/ml | 42 | 520.6 ± 332.2 pg/ml |
| Shuval-Sudai | 2003 | Israel | Mean ± SD | 96 | 284.7 ± 19.2 pg/ml | 37 | 283.1 ± 11.3 pg/ml |
| Trimarchi | 2004 | Argentina | Mean ± SD | 8 | 225.4 ± 111.9 | 21 | 707.9 ± 258.3 pg/ml |
| Oijen | 2004 | Netherlands | Mean ± SD | 29 | 1033 ± 741 pmol/l | 60 | 971 ± 717 pmol/l |
| Sarari | 2008 | Palestine | Mean ± SEM | 43 | 264.5 ± 22.9 pg/ml | 17 | 419.7 ± 39.8 pg/ml |
| Stettin | 2008 | Germany | Mean ± SD | 69 | 274 ± 95.3 pmol/l | 21 | 333 ± 164 pmol/l |
| Kakehasi | 2009 | Brazil | Mean ± SD | 34 | 546.3 ± 427.4 pg/ml | 27 | 576.3 ± 483.3 pg/ml |
| Gerig | 2013 | Switzerland | Mean ± SD | 85 | 274 ± 92 ng/l | 319 | 272 ± 121 ng/l |
| Ulasoglu | 2019 | Turkey | Mean ± SD | 213 | 276.5 ± 134 pg/ml | 76 | 338.4 ± 160.8 pg/ml |
| Surmeli | 2019 | Turkey | Median (IQR) | 43 | 369 (198-498) pg/ml | 211 | 327 (216-616) pg/ml |
| Soyocak | 2021 | Turkey | Median (IQR) | 31 | 293 (237-372) pg/ml | 19 | 420 (338-459) pg/ml |
| **Folate** |  |  |  |  |  |  |  |
| Tamura | 2002 | Japan | Mean ± SD | 57 | 6.2 ± 2.1 ng/ml | 36 | 7.4 ± 2.8 ng/ml |
| Cenerelli | 2002 | Italy | Mean ± SD | 31 | 15.4 ± 5.7 nmol/l | 42 | 16.6 ± 5.2 nmol/l |
| Shuval-Sudai | 2003 | Israel | Mean ± SD | 96 | 5.7 ± 0.2 ng/ml | 37 | 6.9 ± 0.5 ng/ml |
| Stettin | 2008 | Germany | Mean ± SD | 69 | 23.7 ± 7.2 nmol/l | 21 | 24.0 ± 8.2 nmol/l |
| Gerig | 2013 | Switzerland | Mean ± SD | 85 | 5.6 ± 3.1 μg/l | 319 | 5.5 ± 3.2 μg/l |
| Ulasoglu | 2019 | Turkey | Mean ± SD | 213 | 8.3 ± 3.2 ng/dl | 76 | 10.9 ± 4.0 ng/dl |
| Surmeli | 2019 | Turkey | Median (IQR) | 43 | 8.3 (6.1-10.9) ng/ml | 211 | 8.0 (5.7-11.4) ng/ml |
| Soyocak | 2021 | Turkey | Mean ± SD | 31 | 8.6 ± 3.7 ng/ml | 19 | 9.2 ± 3.1 ng/ml |
| **Vitamin C** |  |  |  |  |  |  |  |
| Banerjee | 1994 | UK | Median (range) | 19 | 3.0 (0.6-19.8) μg/ml | 10 | 8.0 (0.9-23.1) μg/ml |
| Rokka | 1995 | USA | Median (range) | 58 | 12.4 (2.9-29.4) μg/ml | 30 | 14.0 (9.0-16.2) μg/ml |
| Webb | 1997 | Australia | Mean (95%CI) | 666 | 49.6 (49.4-53.7) μmol/l | 737 | 51.5 (47.4-51.9) μmol/l |
| Phull | 1998 | UK | Median (IQR) | 25 | 64.9 (60.3-76.5) μmol/l | 18 | 67.0 (57.8-77.4) μmol/l |
| Rokkas | 1999 | Greece | Median (range) | 30 | 18.7 (3.7-35.6) mg/l | 10 | 18.2 (14.1-30.3) mg/l |
| Jarosz(1) | 2000 | Poland | Median (range) | 21 | 6.3 (3.2-12.4) μg/ml | 17 | 6.8 (3.1-12.8) μg/ml |
| Jarosz(2) | 2000 | Poland | Median (range) | 31 | 3.7 (1.8-8.0) μg/ml | 21 | 4.6 (1.9-11.3) μg/ml |
| Toyonaga | 2000 | Japan | Mean ± SD | 37 | 5.2 ± 2.0 μg/ml | 30 | 5.2 ± 2.3 μg/ml |
| Woodward | 2001 | UK | Mean (95%CI) | 765 | 4.2 (0.7-26.3) μg/ml | 403 | 6.5 (1.3-31.8) μg/ml |
| Everett | 2001 | UK | Median (IQR) | 85 | 7.2 (3.6-10.6) μg/ml | 39 | 9.8 (6.4-12.5) μg/ml |
| Annibale | 2003 | Italy | Median (range) | 30 | 11.0 (5.9-21.4) μg/ml | 13 | 19.0 (5.3-49.0) μg/ml |
| Capurso | 2003 | Italy | Median (range) | 32 | 10.0 (2.6-32.2) μg/ml | 13 | 7.4 (0.5-75.0) μg/ml |
| Simon | 2003 | USA | Median (range) | 2189 | 0.71 (0.001-2.85) mg/dl | 4557 | 0.75 (0.001-2.95) mg/dl |
| Khanzode | 2003 | India | Mean ± SEM | 37 | 0.57 ± 0.009 μmol/l | 40 | 0.65 ± 0.010 μmol/l |
| **Vitamin D** |  |  |  |  |  |  |  |
| Antico | 2012 | Italy | Mean ± SD | 21 | 11.3 ± 8.4 ng/ml | 163 | 21.3 ± 12.2 ng/ml |
| Gerig | 2013 | Switzerland | Mean ± SD | 85 | 49 ± 30 nmol/l | 319 | 52 ± 29 nmol/l |
| Han | 2019 | China | Mean ± SD | 496 | 17.0 ± 6.9 ng/ml | 257 | 19.2 ± 8 ng/ml |
| Surmeli | 2019 | Turkey | Median (IQR) | 43 | 9.0 (5.8-17.1) ng/ml | 211 | 13.6 (7.5-22.7) ng/ml |
| Assaad | 2019 | Lebanon | Mean ± SD | 225 | 18.0 ± 7.2 ng/ml | 235 | 30.7 ± 15.7 ng/ml |
| Gao | 2020 | China | Median (IQR) | 2113 | 26.3 (21.1-28.2) ng/ml | 4783 | 26.7 (22.5-28.6) ng/ml |
| Shafrir | 2021 | Israel | Mean ± SD | 75640 | 18.6 ± 9.8 ng/ml | 74843 | 20.1 ± 9.4 ng/ml |
| **Vitamin E** |  |  |  |  |  |  |  |
| Phull | 1998 | UK | Median (IQR) | 25 | 11.1 (7.7-12.2) mg/l | 18 | 10.0 (6.9-12.0) mg/l |
| Zhang | 2000 | UK | Median (IQR) | 41 | 21.4 (14.6-32.2) μmol/l | 27 | 23.1 (14.9-27.2) μmol/l |
| Toyonaga | 2000 | Japan | Mean ± SD | 37 | 1.2 ± 0.4 mg/dl | 30 | 1.1 ± 0.3 mg/dl |

SD: standard deviation; SEM: standard error of mean; IQR: interquartile range; CI: confidence Interval;
