# Supplementary material for: Vitamins and Helicobacter pylori: An Updated Comprehensive Meta-Analysis and Systematic Review
Source: Front Nutr. 2022 Jan 18;8:781333. doi: 10.3389/fnut.2021.781333 (PMC8805086; doi:10.3389/fnut.2021.781333)
Supplement: Supplementary file 7 [file Table_3.docx]

Supplementary Table 3 Original data of the studies comparing serum vitamin level between the successful HP eradication groups and the failed groups

| Study | Year | Area | Estimates presentation | No. of successful groups | Original data of successful groups | No. of failed groups | Original data of failed groups |
| --- | --- | --- | --- | --- | --- | --- | --- |
| **Vitamin D** |  |  |  |  |  |  |  |
| Yildirim | 2017 | Turkey | Mean ± SD | 170 | 19.0 ± 8.1 ng/ml | 50 | 9.1 ± 4.7 ng/ml |
| Shahawy | 2018 | Egypt | Mean ± SD | 105 | 27.4 ± 7.1 ng/ml | 45 | 14.7 ± 4.5 ng/ml |
| Magsi | 2021 | Pakistan | Mean ± SD | 88 | 31.0 ± 7.8 ng/ml | 36 | 18.9 ± 5.6 ng/ml |
| Shatla | 2021 | Saudi Arabia | Mean ± SD | 109 | 28.1 ± 8.1 ng/ml | 42 | 13.5 ± 6.4 ng/ml |
| Shafrir | 2021 | Israel | Mean ± SD | 45821 | 19.3 ± 9.8 ng/ml | 29722 | 18.6 ± 9.6 ng/ml |
