# Supplementary material for: Vitamins and Helicobacter pylori: An Updated Comprehensive Meta-Analysis and Systematic Review
Source: Front Nutr. 2022 Jan 18;8:781333. doi: 10.3389/fnut.2021.781333 (PMC8805086; doi:10.3389/fnut.2021.781333)
Supplement: Supplementary file 8 [file Table_4.docx]

Supplementary Table 4 Original data of the studies comparing serum vitamin levels before and after HP eradication therapy

| Study | Year | Area | Estimates presentation | No. of self-controlled groups | Original data before eradication therapy | Original data after eradication therapy |
| --- | --- | --- | --- | --- | --- | --- |
| **Vitamin B_12_** |  |  |  |  |  |  |
| Kaptan | 2000 | Turkey | Mean ± SD | 31 | 63 ± 30 pmol/l | 233 ± 38 pmol/l |
| Serin | 2002 | Turkey | Mean ± SD | 65 | 145 ± 28 pg/ml | 232 ± 39 pg/ml |
| Ozer | 2005 | Turkey | Mean ± SD | 41 | 210 ± 97 pg/ml | 237 ± 39 pg/ml |
| Marino(1) | 2007 | Brazil | Mean ± SD | 59 | 145.5 ± 48.7 pmol/l | 209.8 ± 87.1 pmol/l |
| Marino(2) | 2007 | Brazil | Mean ± SD | 59 | 145.5 ± 48.7 pmol/l | 271.2 ± 140.8 pmol/l |
| **Folate** |  |  |  |  |  |  |
| Kaptan | 2000 | Turkey | Mean ± SD | 31 | 25 ± 5 nmol/l | 20 ± 6 nmol/l |
| Ozer | 2005 | Turkey | Mean ± SD | 41 | 5.6 ± 2.6 ng/ml | 6.0 ± 2.4 ng/ml |
| **Vitamin C** |  |  |  |  |  |  |
| Banerjee(1) | 1994 | UK | Median (range) | 11 | 2.7 (0.63-9.6) μg/ml | 2.7 (1.5-14.7) μg/ml |
| Banerjee(2) | 1994 | UK | Median (range) | 11 | 2.7 (0.63-9.6) μg/ml | 6.1 (0.4-13.8) μg/ml |
| Annibale(1) | 2003 | Italy | Median (range) | 5 | 18.1 (14.8-20.7) μg/ml | 9.0 (7.4-32.2) μg/ml |
| Annibale(2) | 2003 | Italy | Median (range) | 5 | 7.0 (6.5-8.2) μg/ml | 6.0 (3.0-8.6) μg/ml |
