# Supplementary material for: Vitamins and Helicobacter pylori: An Updated Comprehensive Meta-Analysis and Systematic Review
Source: Front Nutr. 2022 Jan 18;8:781333. doi: 10.3389/fnut.2021.781333 (PMC8805086; doi:10.3389/fnut.2021.781333)
Supplement: Supplementary file 9 [file Table_5.docx]

Supplementary Table 5 Original data of the studies focusing on the effect of vitamin supplement on HP eradication rate

| Study | Year | Area | Type of vitamin supplement | No. of supplement groups | No. of successful eradication of supplement groups | No. of control groups | No. of successful eradication rate of control groups |
| --- | --- | --- | --- | --- | --- | --- | --- |
| **Per-protocol Analysis** | | | | | | | |
| Chuang | 2002 | China | Vitamin C and E | 50 | 22 | 45 | 29 |
| Sezikli | 2009 | Turkey | Vitamin C and E | 78 | 73 | 75 | 48 |
| Sezikli | 2011 | Turkey | Vitamin C and E | 77 | 51 | 38 | 17 |
| Sezikli | 2012 | Turkey | Vitamin C and E | 157 | 132 | 38 | 18 |
| Demirci(1) | 2015 | Turkey | Vitamin C and E | 84 | 60 | 84 | 63 |
| Demirci(2) | 2015 | Turkey | Vitamin C and E | 91 | 76 | 89 | 72 |
| Chuang | 2007 | China | Vitamin C | 58 | 52 | 51 | 37 |
| Zojaji | 2009 | Iran | Vitamin C | 141 | 117 | 140 | 79 |
| **Intention-to-treat Analysis** | | | | | | | |
| Chuang | 2002 | China | Vitamin C and E | 55 | 22 | 49 | 29 |
| Everett | 2002 | UK | Vitamin C and E | 24 | 19 | 25 | 17 |
| Sezikli | 2009 | Turkey | Vitamin C and E | 80 | 73 | 80 | 48 |
| Sezikli | 2011 | Turkey | Vitamin C and E | 80 | 51 | 40 | 17 |
| Sezikli | 2012 | Turkey | Vitamin C and E | 160 | 132 | 40 | 18 |
| Demirci(1) | 2015 | Turkey | Vitamin C and E | 100 | 60 | 100 | 63 |
| Demirci(2) | 2015 | Turkey | Vitamin C and E | 100 | 76 | 100 | 72 |
| Kockar | 2001 | Turkey | Vitamin C | 30 | 20 | 30 | 15 |
| Chuang | 2007 | China | Vitamin C | 61 | 52 | 45 | 37 |
| Zojaji | 2009 | Iran | Vitamin C | 150 | 117 | 162 | 79 |
