# Supplementary material for: Vitamins and Helicobacter pylori: An Updated Comprehensive Meta-Analysis and Systematic Review
Source: Front Nutr. 2022 Jan 18;8:781333. doi: 10.3389/fnut.2021.781333 (PMC8805086; doi:10.3389/fnut.2021.781333)
Supplement: Supplementary file 10 [file Table_6.DOCX]

Supplementary Table 6. Assessment of risk bias of the RCT studies included in the meta-analysis

|  | Selection bias | Selection bias | Performance bias | Detection bias | Attrition bias | Reporting bias |
| --- | --- | --- | --- | --- | --- | --- |
| Study | Random sequence  generation | Allocation concealment | Blinding of participants and personnel | Blinding of outcome assessment | Incomplete outcome data | Selective reporting |
| Chuang 2002 | Unclear | Low | Unclear | Low | Low | Low |
| Everett 2002 | Unclear | Low | Low | Low | Low | Low |
| Sezikli 2009 | Unclear | Low | High | Low | Low | Low |
| Sezikli 2011 | Unclear | Low | Unclear | Low | Low | Low |
| Sezikli 2012 | Unclear | Low | High | Low | Low | Low |
| Demirci 2015 | Unclear | Low | Unclear | Low | Low | Low |
| Kockar 2001 | Unclear | Low | Unclear | Low | Low | Low |
| Chuang 2007 | Unclear | Low | Unclear | Low | Low | Low |
| Zojaji 2009 | Unclear | Low | Unclear | Low | Low | Low |
